# Adjuvant Corticosteroid Therapy in Hepatosplenic Candidiasis-Related Iris

**DOI:** 10.4084/MJHID.2012.018

**Published:** 2012-03-13

**Authors:** Cengiz Bayram, Ali Fettah, Nese Yarali, Abdurrahman Kara, Fatih Mehmet Azik, Betul Tavil, Bahattin Tunc

**Affiliations:** Department of Pediatric Hematology, Ankara Children’s Hematology and Oncology Hospital, Ankara, Turkey

## Abstract

Candida infections are the most frequent infections in neutropenic patients. Hepatosplenic candidiasis (HSC) is a part of disseminated Candida infection that occurs most commonly in patients with hematologic malignancies treated with chemotherapy and requires protracted antifungal therapy. During invasive mycosis with rapid resolution of immunosuppression, immune reconstitution inflammatory syndrome (IRIS) which mimics treatment failure, drug toxicity or breakthrough infections may occur. Manifestation period, histopathologic findings and favorable effect of steroids to its inflammatory symptoms strongly suggest that HSC belongs to the invasive fungal infection induced IRIS. We present a child with B cell-acute lymphoblastic leukemia who developed HSC and addition of corticosteroid therapy to antifungal treatment achieved rapid resolution of the clinical symptoms and laboratory findings.

## Introduction

Hepatosplenic candidiasis, also called “chronic disseminated candidiasis” is a part of severe invasive *Candida* infection with prominent involvement of the liver, spleen and occasionally the kidneys and other organs.^1^,^2^ During rapid resolution of immunosuppressive conditions, immune reconstitution inflammatory syndrome (IRIS), an exaggerated inflammatory response to some opportunistic microorganisms such as *Mycobacterium tuberculosis, Histoplasma capsulatum, Cryptococcus neoformans, Aspergillus* species may occur.^3^–^5^ Hepatosplenic candidiasis share several features with IRIS related with invasive fungal infections such as occurrence during neutrophil recovery, histopathologic findings and the beneficial effect of steroids to its inflammatory symptoms. These features strongly suggest that HSC belongs to the invasive fungal infection induced IRIS.^6^

Here, we report a case of HSC developing an exaggerated inflammatory response during neutrophil recovery after leukemia induction therapy. Addition of corticosteroid therapy to antifungal therapy has achieved a rapid and complete resolution of clinical and laboratory findings.

### Case Report

**Figure 1:**
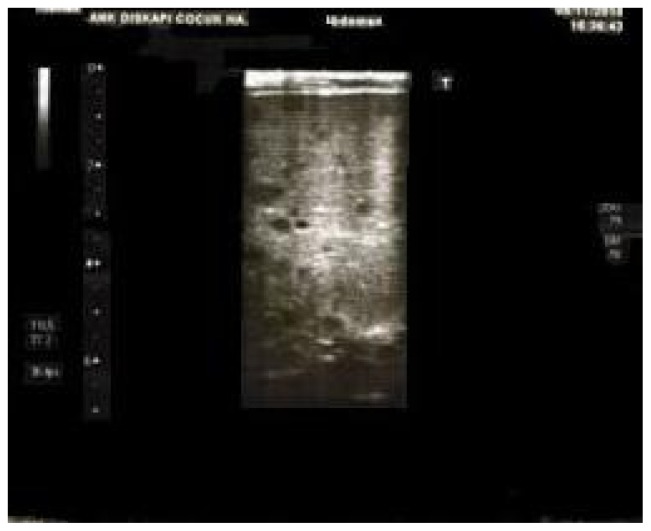
The sonographic appearance of multiple hypoechoic millimetric hepatic nodules.

**Figure 2:**
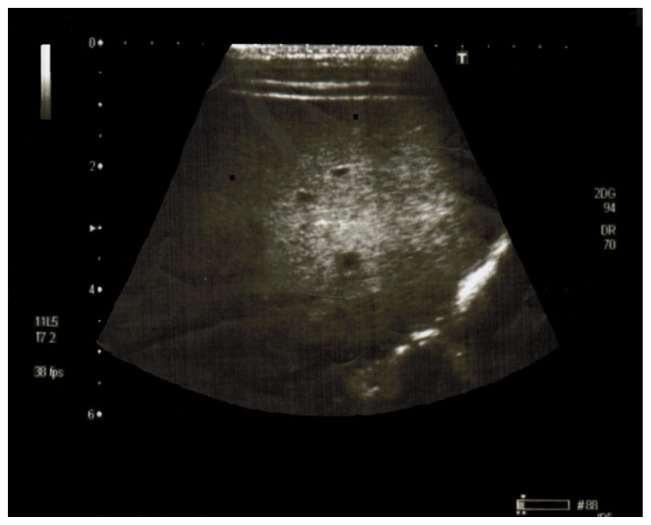
Hepatic ultrasound image 30 days after initiation of corticosteroid therapy.

16-month old boy diagnosed with standard-risk precursor B cell-acute lymphoblastic leukemia was initiated induction therapy of ALL BFM 95 treatment protocol which consisted of prednisone (60 mg/m^2^/day for day 1–28, and tapering for day 29–37), daunorubicin (30 mg/m^2^/day for day 8 and 15), L-asparaginase (5,000 U/m^2^/day for day 12, 15, 18, 21, 24, 27, 30 and 33), vincristine (1,5 mg/m^2^/day for day 8, 15, 22 and 29) and intratechal methotrexate (at age-adjusted dose for day 1, 12 and 33).^6^ On day 15, bone marrow aspiration revealed <5% blast, slightly decreased cellularity with lymphocyte predominance. On the 17^th^ day of therapy, he developed febrile neutropenia and imipenem/cilastatin (60 mg/kg/day) and vancomycin (40 mg/kg/day) was commenced. On day 20, fluconazole (loading dose 12 mg/kg/day iv, followed by 6 mg/kg/day q 24 hr iv) was added to therapy because of persistent fever and mild oral moniliasis. On day 22, he developed tachypnea; at physical examination rales were present. Despite a presumptive diagnosis of pneumonia, computed tomography (CT) of thorax and abdominal ultrasonography were unremarkable. Blood and urine cultures and galactomannan antigen were all negative. Although oral moniliasis resolved, fever and rales persisted and repeated thorax CT demonstrated several ground grass areas and a 5mm nodular lesion surrounded by ground-glass opacity. For a possible Aspergillus infection, fluconazole therapy was shifted to voriconazole (loading dose 6 mg/kg/dose 2 doses, maintenance dose 4 mg/kg/dose iv 2 doses) on day 25. His condition improved shortly thereafter. Induction chemotherapy was completed along with antibiotic and antifungal treatment despite persistence of fever. In the interim, his leukocyte count, serum alkaline phosphatase (ALP), serum γ-glutamyl transferase (GGT) and serum C-reactive protein (CRP) levels began to rise, and reached their top values including leukocyte count of 36.1×10^9^/L, ALP of 1096 U/L (normal range, 145–420 U/L), GGT of 674 U/L (normal range, 5–32 U/L) and CRP of 23 mg/dl (normal range, 0.08–1.12 mg/dl). Peripheral blood smear revealed neutrophilia without any blast. At abdominal ultrasonography multiple hypoechoic millimetric hepatic nodules were noted (Figure 1). Despite his good clinical condition, persistence of fever, elevated ALP and CRP levels and hypoechoic ultrasonographic images in the liver, led us to the diagnosis of possible HSC.^7^ Although the use of antibacterial, antifungal drugs and chemotherapeutics in our patient might have caused hepatotoxicity and therefore mimic laboratory findings of HSC, combination of clinical, laboratory and radiological findings following neutrophil recovery led us to the diagnosis of HSC. Dexamethasone (0,5 mg/kg) was added to voriconazole therapy. Fever disappeared in three days period and leukocyte count, serum ALP, GGT and CRP levels were normalized within 7 days. Corticosteroid therapy was, thereafter, tapered in a 7 days period and stopped. Hypoechoic ultrasound images in the liver disappeared 30 days after initiation of corticosteroid therapy (Figure 2). Antifungal therapy in combination with chemotherapy was continued for three months. He is currently in remission and receiving maintenance therapy of BFM.

### Discussion

Hepatosplenic candidiasis has been reported in cancer patients particularly with acute leukemia during neutropenia.^8^–^10^ It is often clinically apparent during neutrophil recovery.^9^ The frequency of HSC has been reported between 3–29% depending on the diagnostic criteria.^10^ Although not specific for HSC, young age, neutropenia (absolute neutrophil count <500 μL) lasting more than 15 days, and the use of prophylactic quinolones have been reported to be independent risk factors for both acute and chronic invasive candidiasis.^10^ It has been hypothesized that HSC develops as a result of prolonged neutropenia and gastrointestinal mucosal damage in the context of immune dysregulation due to defective coordination of Th1/Th2 response in conjunction with other immune imbalance.^3^,^10^ Recently Th1/Th17 pathway has been implicated in the pathogenesis of fungal infection related IRIS.^11^ There has been increased recognition of the role of endogenous levels of certain cytokines such as IL-10, IL-17, IL-23 and some immunomodulators as likely contributing factors in the pathogenesis of systemic candidal infection.^6^,^10^,^11^

A diagnosis of HSC should be suspected with fever unresponsive to broad-spectrum antibiotics during neutropenic stage and following its recovery, and the presence of symptoms and physical findings including upper abdominal pain, tenderness, jaundice, and hepatosplenomegaly. Increased serum liver function tests, abnormal hepatosplenic imaging at CT or ultrasonography and histopathologic evidence of Candida spp. in the biopsy specimens make the diagnosis of HSC.^10^ Furthermore, clinical/laboratory evidence of other infections and/or clinical conditions such as progressive infection, drug toxicity or breakthrough infections should be excluded.

Amphotericine B is accepted as the initial agent of choice for HSC. Alternative options include the use of azoles particularly fluconazole, voriconazole and echinocandins.^12^,^13^ Therapy should be continued until calcification or resolution of lesions, particularly in patients receiving continued chemotherapy or immunosuppression.^12^ Hepatosplenic candidiasis requires several months of antifungal therapy, thus interrupt the treatment of underlying leukemia. Delay in the treatment of leukemia may affect the course and survival of these patients. As recently reported, addition of corticosteroid therapy to antifungal therapy in patients with HSC related IRIS has found to be associated with disappearance of clinical symptoms, radiologic abnormalities and inflammatory markers.^6^,^14^ It was supposed that corticosteroids might adjust the paradoxical immune response by modulating both mononuclear phagocyte function and T-cell activation.^6^ In our case, we observed that adjuvant corticosteroid therapy, in addition to antifungal treatment, provided a rapid and complete resolution of clinical and laboratory and subsequently radiological findings.

In conclusion, HSC-related IRIS should be suspected in febrile immune compromised patients especially during neutrophil recovery with exaggerated inflammatory response and persistent symptoms despite receiving antifungal therapy. It should be kept in mind that, addition corticosteroid therapy to appropriate antifungal treatment may result in prompt resolution of clinical and laboratory findings.
